# The GPCR Antagonistic Drug CM-20 Stimulates Mitochondrial Activity in Human RPE Cells

**DOI:** 10.2174/1874091X-v16-e2206270

**Published:** 2022-08-22

**Authors:** Qing Chang, Siquan Chen, Tahua Yang

**Affiliations:** 1 University of Illinois Technology Innovation Lab and Argos Vision Inc., Chicago, United States; 2 Cellular Screening Center, The University of Chicago, Chicago, United States

**Keywords:** GPCR, Mitochondria, Polypharmacology, Multi-target drug, RPE, Oxidative stress, Age-related macular degeneration (AMD)

## Abstract

**Background:**

Mitochondrial dysfunction in retinal pigment epithelium (RPE) is a pathogenic factor in age-related macular degeneration (AMD). Improvement of mitochondrial function may ameliorate RPE bioenergetics status, which may in turn nourish the retinal photoreceptors against degenerative loss.

**Objective:**

The purpose of this study is to examine the G-protein coupled receptor (GPCR) antagonistic drug CM-20 in modulating mitochondrial function in RPE cells.

**Methods:**

Human-derived ARPE-19 cell line was differentiated to improve RPE morphology. Dose response of CM-20 was performed to examine mitochondrial membrane potential (MMP). Secondary validation with multiplexed live-cell mitochondrial imaging was performed. Protection of CM-20 to mitochondria against oxidative stress was detected under co-treatment with hydrogen peroxide.

**Results:**

Treatment with CM-20 elicited a dose-dependent increase of MMP. Multiplexed live-cell mitochondrial imaging showed consistent increase of MMP at an optimal concentration of CM-20 (12.5 μM). MMP was significantly reduced under hydrogen peroxide-induced oxidative stress and treatment with CM-20 showed rescue effects to MMP.

**Conclusion:**

CM-20 increases mitochondrial function and protects mitochondria under oxidative stress. As both GPCRs and mitochondria are potential drug targets, retinal neuroprotective testing of CM-20 is warranted in animal models of retinal degeneration.

## 1. INTRODUCTION

Age-related macular degeneration (AMD) is a leading cause of irreversible vision impairment and loss in the elderly population [1]. AMD occurs in two forms: the neovascular (wet) form and the atrophic (dry) form. Anti-vascular endothelial growth factor therapy is the standard care for the treatment of wet AMD [2]. There is no approved therapy for dry AMD to date. AMD typically initiates with degeneration of retinal pigment epithelial (RPE) cells, followed by degeneration of their supporting cells, the retinal photoreceptors [3]. There is evidence to suggest that RPE cells and photoreceptors are maintained in a symbiotic relationship [4]. Stressed RPE cells not only cause dysfunction and degeneration of themselves per se but also lead to collateral damage of photoreceptors [5]. In etiology, oxidative stress is suggested to play key pathogenic roles in the early stage of AMD [6]. There is an observation of greater loss of both mitochondrial number and content in the RPE layer of AMD eyes than normal aging eyes [7]. Increased mitochondrial DNA damage is also found in the RPE cells of AMD eyes that is correlated with the disease stage [8]. Primary RPE cell cultures derived from AMD eyes exhibited more loss in the capacity of mitochondrial respiration than those derived from normal aging eyes [9].

Our previous work using an unbiased phenotype-based high-throughput chemical screen identified neuroprotective lead compounds in photoreceptors against environmental risk factor (light)-induced oxidative damage and mitochondriainitiated apoptosis [10, 11]. Given the symbiotic relationship between RPE cells and photoreceptors, pharmaceutical drugs that protect both RPE cells and photoreceptors would be more desirable from a therapeutic standpoint. To test this idea and integrate the strategy of polypharmacology using multi-targeting drugs for more effective treatment of complex diseases, including neurodegenerative disorders in the central nervous system [12], we rescreened the photoreceptor neuroprotective lead compounds in cultured RPE cells to see whether positive hits targeting mitochondria can emerge. The GPCR antagonist (CM-20) was identified as a leading hit. CM-20 is an FDA-approved drug for the treatment of hypertension and heart disease. Its pharmacological effect can be attributed to its inhibition of alpha adrenoceptors (ARs) [13]. In the visual system, the blockade of alpha ARs protected the retina in animal models of retinal diseases, such as Stargardt disease, retinal detachment, and diabetic retinopathy [14 – 16]. The underlying mechanisms are not entirely clear. They may involve a decrease in reactive oxygen species (ROS) production and neurotrophic roles, such as increasing the expression of photoreceptor genes [17]. Mitochondria are the major cellular source of ROS production and are vulnerable to damage under oxidative stress [18, 19]. In this report, we extended our previous findings to test the role of CM-20 in modulating mitochondrial function in cultured RPE cells.

## 2. MATERIALS AND METHODS

### 2.1. Cell Culture and Differentiation

Human RPE cell line (ARPE-19) was obtained from Fisher Scientific (Pittsburgh, PA, ATCC CRL2302). Frozen cell stock was thawed in a 37° C water bath. Cultures were established in T75 flasks in a medium containing DMEM + GlutaMAX with 10% FBS and 1% penicillin/streptomycin at 37° C supplied with 5% CO_2_. At confluence, cells were trypsinized with TrypLE Express Enzyme phenol red and harvested for differentiation for two weeks [20].

### 2.2. Compound Treatment

CM-20 was dissolved in methanol at 10 mg/ml. To reduce potential confounding effects caused by growth factors, cellular treatment was performed under a low serum condition (1% FBS). The cells were treated with a serially increased concentration of CM20 (0–85 μM) or a fixed concentration (12.5 μM) for a period of 24–72 h.

### 2.3. Multiplexed Live-cell Mitochondrial Imaging

Mitochondrial membrane potential (MMP) was detected in microplate cultures of dRPE cells using the mitochondrial-specific Image-iT TMRM fluorescent reagent (Thermo Fisher Scientific, Waltham, MA). Essentially, dRPE cells were incubated with a non-quenching concentration (10 nM) of TMRM at 37° C. Fluorescent signals were monitored in real-time with a Sartorius IncuCyte S3 microplate reader (Sartorius, Göttingen, Germany) for 4 h. Controls included cells without TMRM and blanks with or without TMRM. Fluorescent signals, including total fluorescent object area (μm^2^/image), total fluorescent integrated intensity (RCU × μm^2^/image), intensity raw value, total fluorescent cell counts, and mean fluorescent intensity/cell, were calculated using IncuCyte Base software. Fluorescent heat map was generated using Excel under conditional formatting and selection of color scale from different data groups.

### 2.4. Oxidative Stress and Detection of Cell Viability

Microplate cultures of dRPE cells were exposed to hydrogen peroxide (250 μM-1 mM) in serum-free DMEM for 5 hours at 37° C. After that, the supernatant was removed with a liquid handler, and the cells were subject to TMRM staining. Cell viability was measured using CellTiter-Glo luminescent reagent (Promega, Madison, WI) following the manufacturer’s instructional protocol.

### 2.5. Statistical Analysis

Two-tailed student’s *t*-test was used to detect the difference between two sample groups using Excel along with Daniel’s XL toolbox [21]. To examine the difference between more than two sample groups, one-way ANOVA was applied. A *P* value of less than 0.05 was considered significant.

## 3. RESULTS

### 3.1. CM-20 Stimulated Mitochondrial Activity

**Fig. (1).**
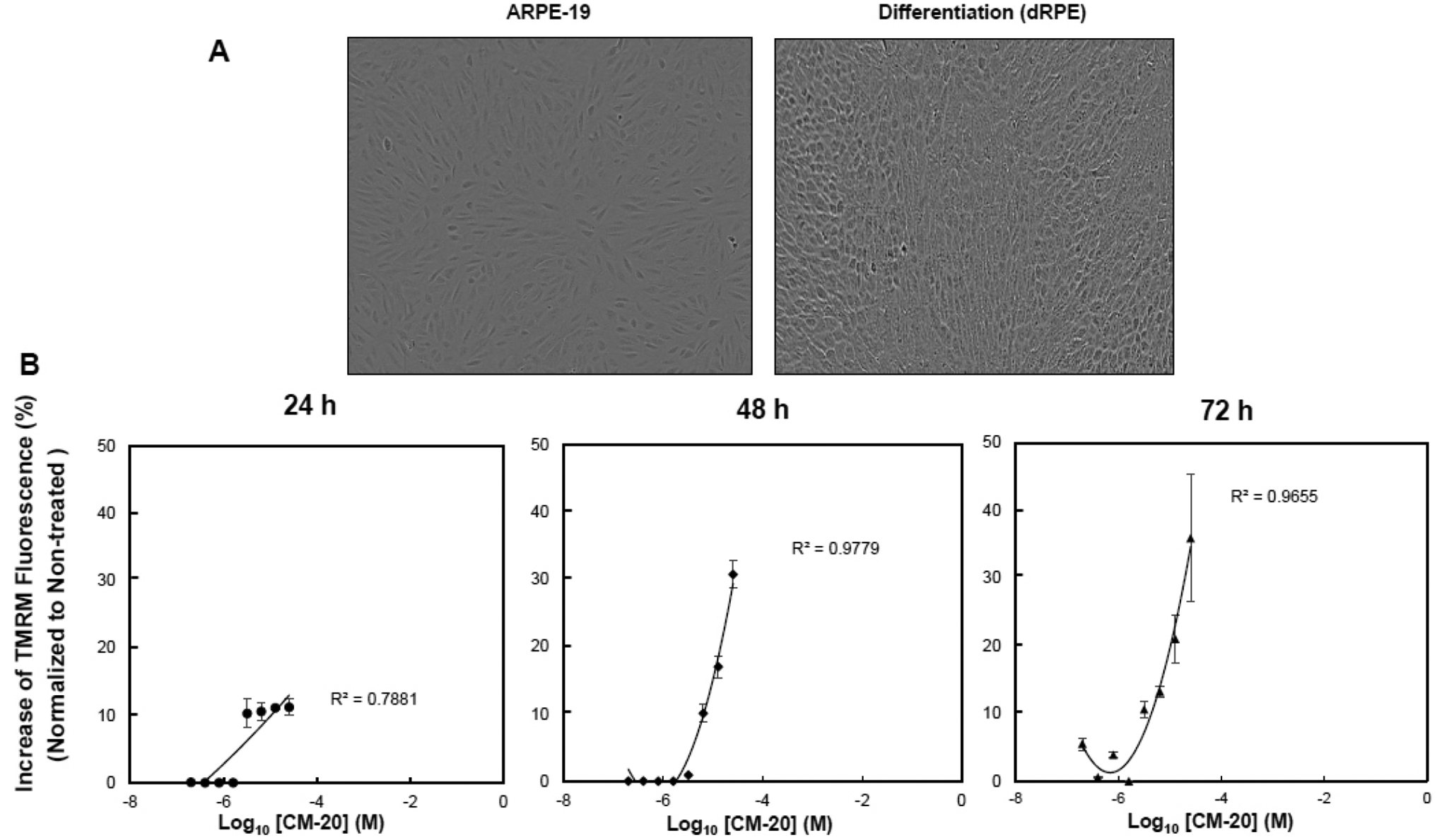
CM-20 treatment elicited a dose-dependent increase of mitochondrial membrane potential (MMP). (**A**) Cultured ARPE-19 cells were differentiated for 2 weeks. Differentiated ARPE-19 cells (dRPE cells, right panel) displayed the cobblestone morphology of native RPE cells. (**B**) Independent microplate cultures of dRPE cells were treated with CM-20 (0–25 μM, n=3) for 24, 48, or 72 h, after which MMP was detected with the mitochondrial-specific fluorescent indicator TMRM. There was a significant correlation between MMP and treatment (R^2^ > 0.7).

To test the bioactivity of CM-20 on mitochondria, we first performed dose-response experiments in differentiated ARPE-19 (dRPE) cells. Following previously published methods [20], we differentiated ARPE-19 cells in order to improve RPE-related morphology. The dRPE cells displayed cobblestone morphology in culture like native RPE cells as compared to the undifferentiated cells, showing elongated fibroblast-like cellular phenotype (Fig. 1A). Cultures of dRPE cells were maintained in a regular medium containing 5% FBS. At confluence, cultures were split and seeded on microplates with equal cell density for 24 h. The medium was then replaced with a low serum medium (1% FBS) to avoid potential confounding effects by growth factors in the serum during compound treatment. Microplate cultures were subjected to one-time treatment with serially increased concentrations of CM-20 for 24–72 h. Mock treatment with solvent (methanol) was used as the control, with all other experimental conditions being identical. Mitochondrial membrane potential (MMP), denoting mitochondrial activity, was detected with the mitochondria-specific fluorescent indicator TMRM [22 – 24]. There was a dose-dependent effect observed on TMRM fluorescence in significant correlation with CM-20 treatment (R^2^ > 0.7) (Fig. 1B). At 24 h, an increase in TMRM fluorescence was observed starting above 3.2 μM compound concentration. At lower compound concentrations (< 3.2 μM), there was no significant change observed in TMRM fluorescence. This concentration-dependent effect in TMRM fluorescence may result from a threshold effect in drug treatment. At a too low drug dose, there would be no treatment effect until an effective dose has been reached. Saturation of TMRM fluorescence was observed at 12.5 μM of compound concentration. A more pronounced increase in TMRM fluorescence was found at 48 h post-treatment. There was no significant loss of TMRM fluorescent signal even at 72 h post-treatment.

### 3.2. Secondary Assays with Multiplexed Live-cell Mitochondrial Imaging

**Fig. (2).**
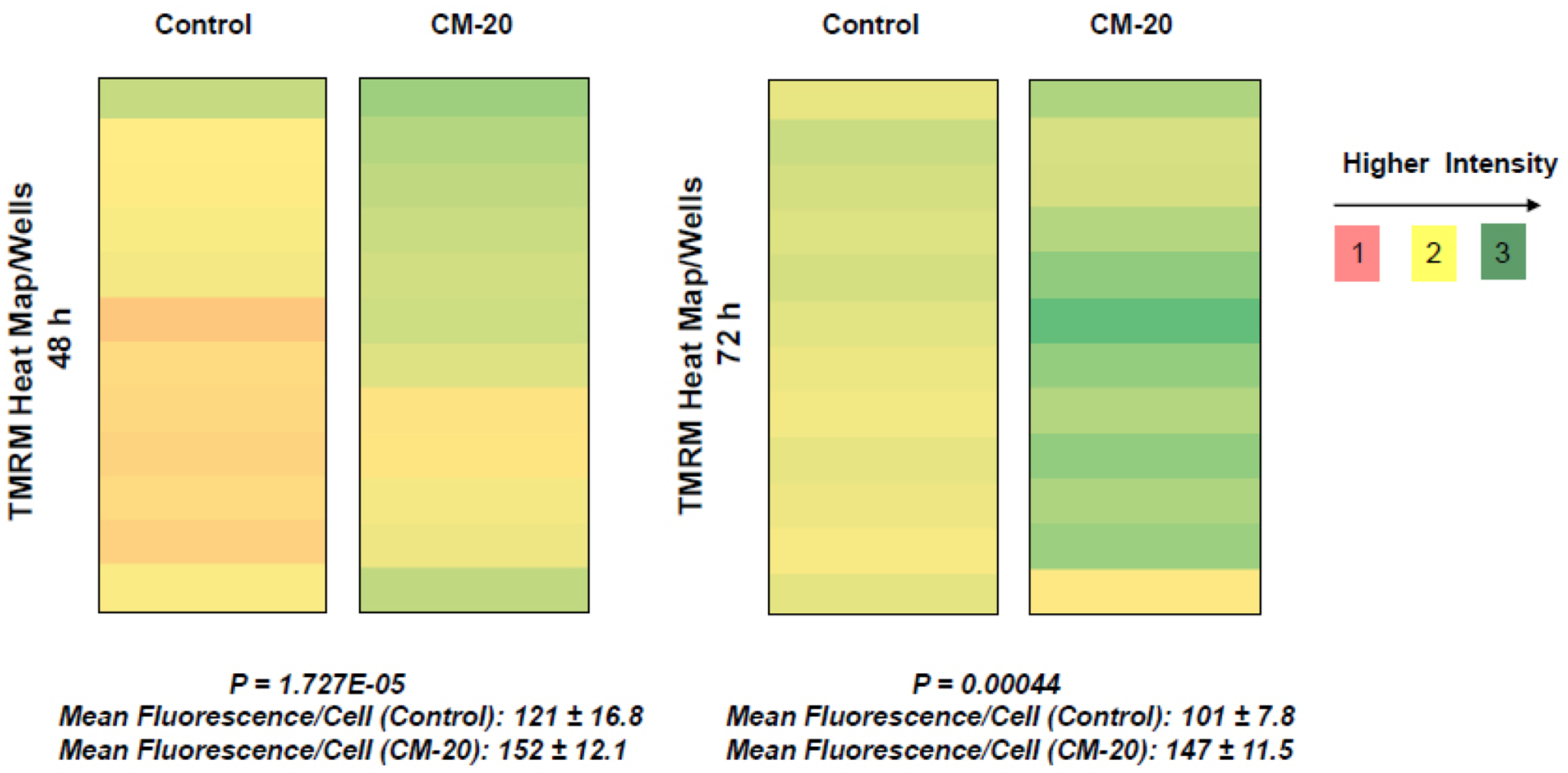
Multiplexed live-cell mitochondrial imaging. Microplate cultures of dRPE cells (n=12) with equal cell density were treated with CM-20 (12.5 μM) or solvent control in low serum (1% FBS) medium for 48 or 72 h. MMP was detected with TMRM. Shown is the heat map of TMRM fluorescence/well and scales of fluorescent intensity. Mean TMRM fluorescence/cell ± SD was significantly higher under treatment with CM-20 at 48 h (*P* = *1.72E*-05) and 72 h (*P* = *0.00044*) compared to solvent control.

**Fig. (3).**
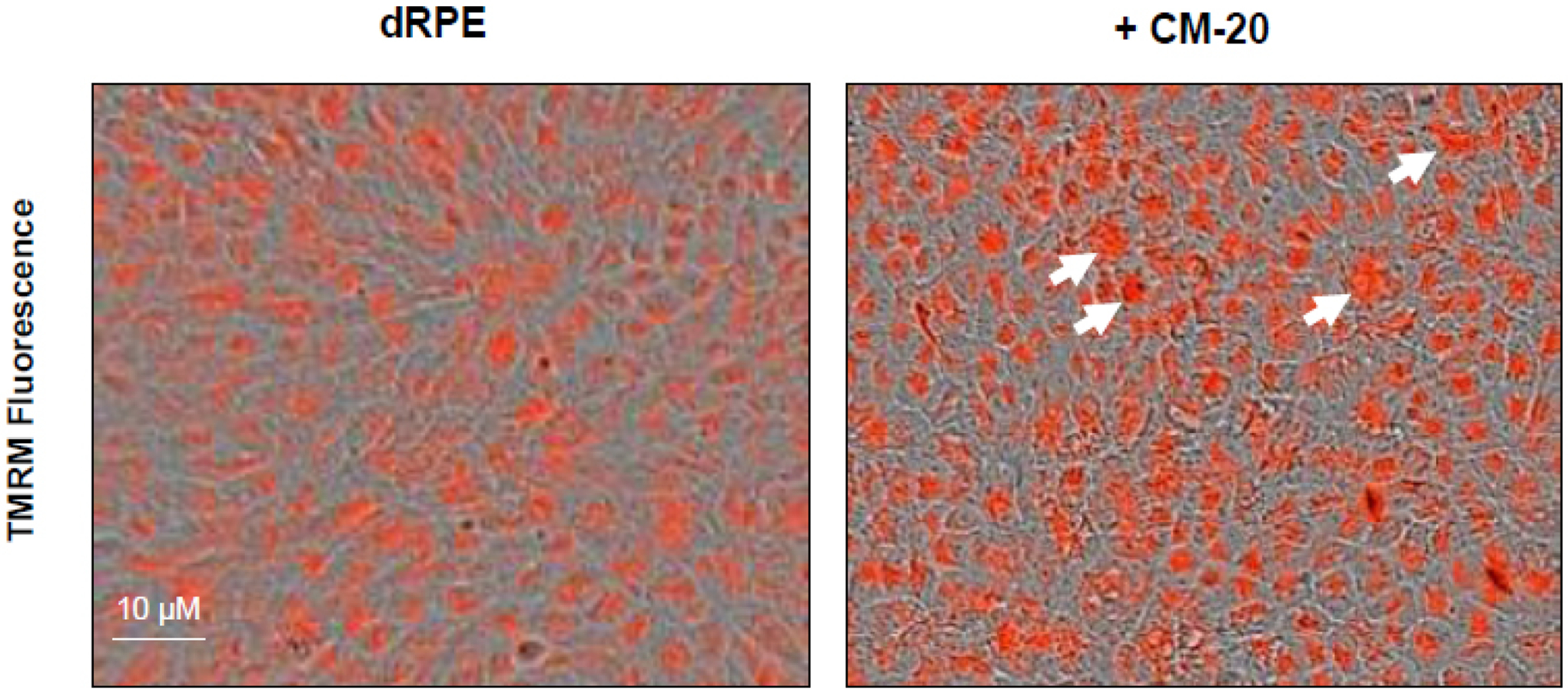
CM-20 treatment increased MMP fluorescence. dRPE cells were treated with CM-20 (12.5 μM) for 48 h, stained with TMRM, and imaged. Treatment with CM20 showed a stronger and denser fluorescent signal of TMRM in virtually all individual cells in the visual field (white arrows) than the solvent control.

To further evaluate the effect of CM-20 on mitochondrial targeting, we conducted multiplexed live-cell mitochondrial imaging of MMP in dRPE cells. The multiplexed assay format significantly increased the robustness, reliability, data output, and power of statistical analysis. Microplate cultures of dRPE cells were treated with CM-20 (12.5 μM) for 24 h to 72 h, followed by TMRM staining to detect MMP. Overall TMRM fluorescence from multiplexed microplate cultures appeared higher in signal intensity under treatment with CM-20 as compared to that of non-treated control at 48 h and 72 h (Fig. 2). The data were further analyzed to reflect the mean TMRM fluorescence at the individual cell level. In this regard, mean TMRM fluorescence/cell showed high significance under treatment with CM-20 at 48 h and 72 h. The significant level (*P*-value) seemed to drop at 72 h, which could result from the decline of compound efficacy overtime. We also conducted a direct microscopic examination of TMRM fluorescence. Fluorescent intensity of TMRM under treatment with CM20 was much higher and denser in individual dRPE cells (Fig. 3). In summary, the live-cell mitochondrial imaging data are consistent with the findings from dose-response experiments, supporting the ability of CM-20 to stimulate mitochondrial function.

### 3.3. Oxidative Stress Damaged Mitochondria but did not Cause Cell Death

**Fig. (4).**
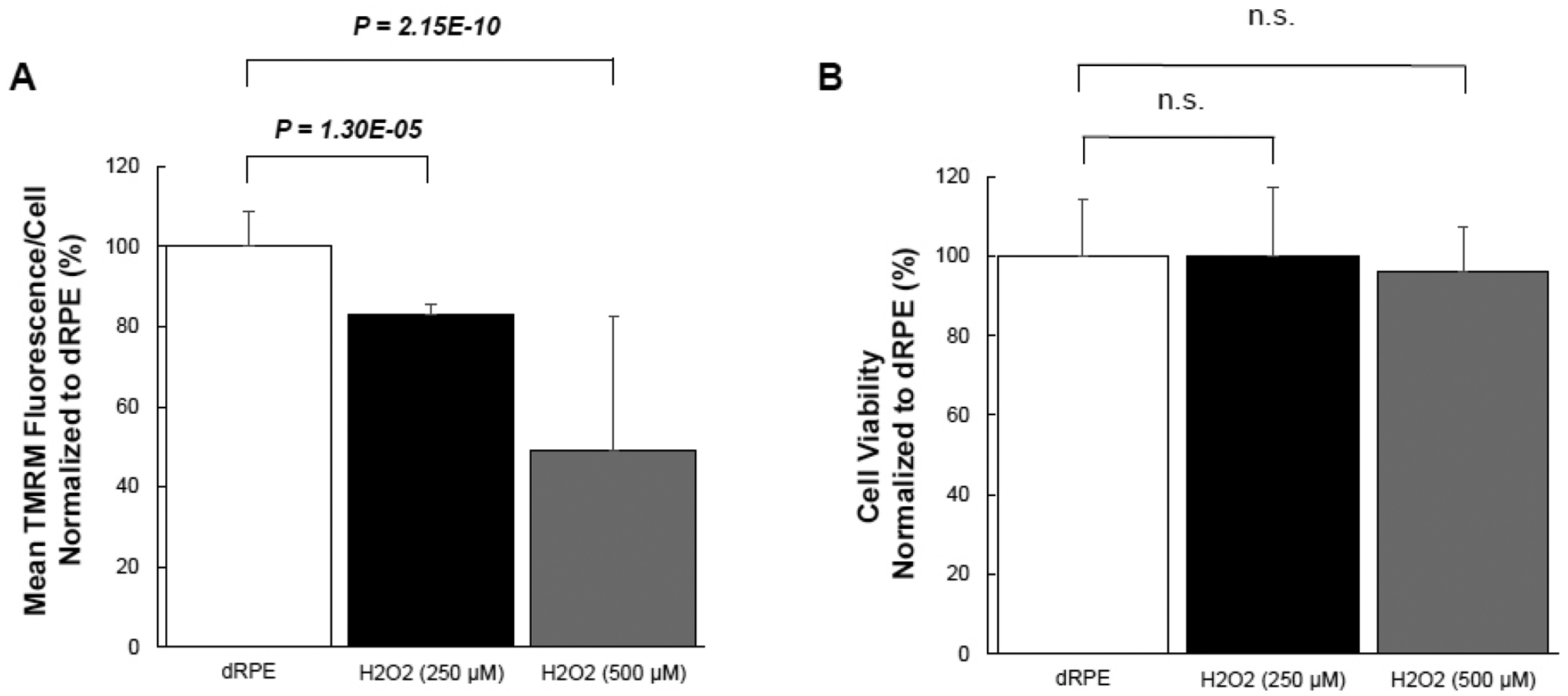
Oxidative stress caused loss of MMP but not cell death. (**A)** dRPE cells were pretreated with CM-20 (12.5 μM, n=4) for 48 h, followed by exposure to hydrogen peroxide (250 μM or 500 μM) for 5 h. There was a significant loss of MMP following exposure to hydrogen peroxide. (**B**) The same cell samples were subject to detection of cell viability. There was no significant difference in cell viability with or without exposure to hydrogen peroxide.

Given the effect of CM-20 on stimulating mitochondrial activity, we next examined whether CM-20 could protect mitochondria from damage under oxidative stress. We first tested the sensitivity of mitochondria under oxidative stress. Exposure of dRPE cells to the retina-generated endogenous oxidative stressor (hydrogen peroxide) caused a significant loss of MMP (Fig. 4A). To know whether oxidative stress-induced mitochondrial damage is associated with cell death, we detected cell viability in the same microplate samples. Surprisingly, there was no significant loss of cell viability (Fig. 4B).

### 3.4. CM-20 Protected Mitochondria under Oxidative Stress

**Fig. (5).**
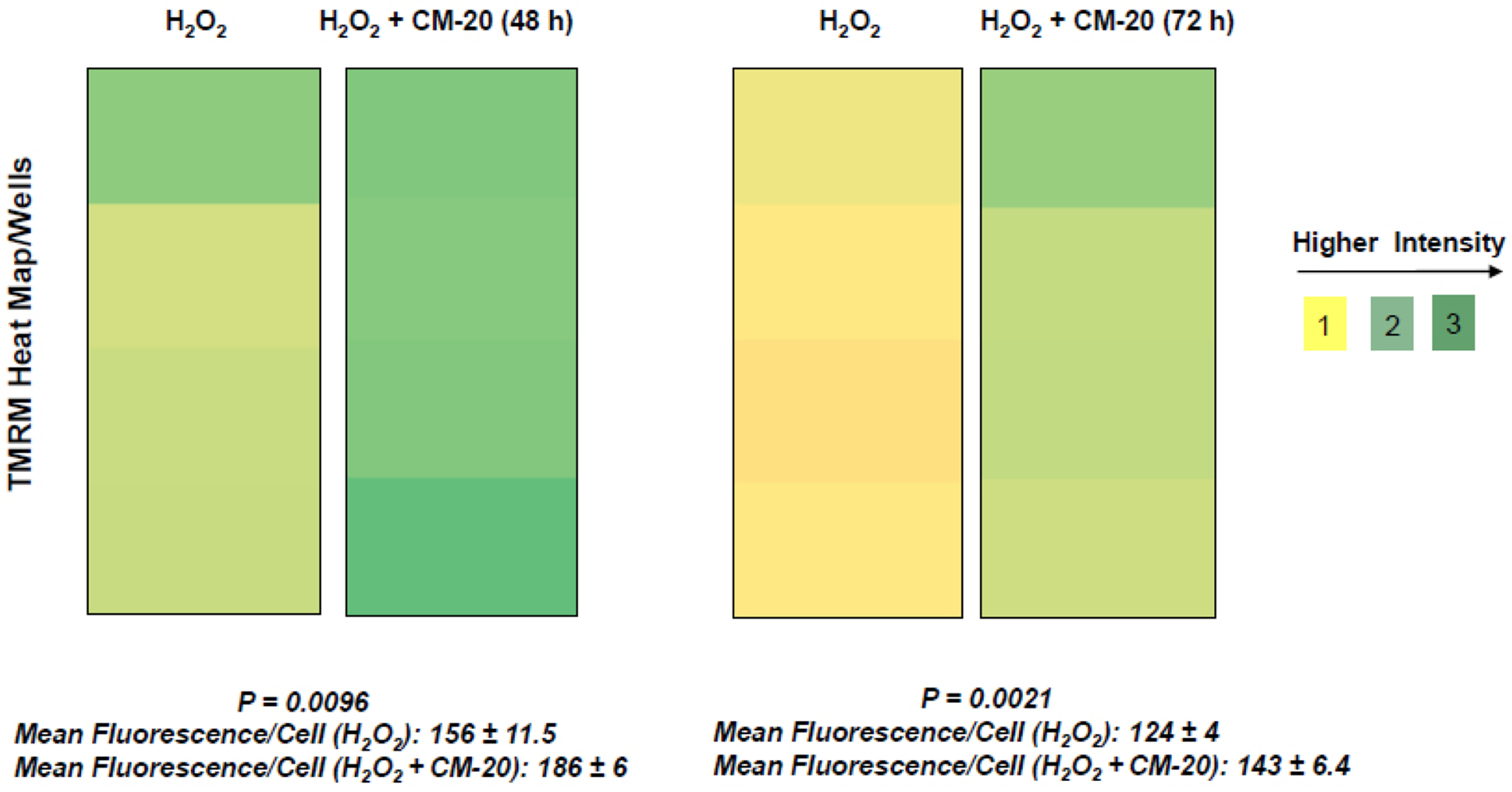
CM-20 rescued the loss of MMP under oxidative stress. Microplate cultures of dRPE cells were treated with CM-20 (12.5 μM, n=4) for 48 h or 72 h, followed by exposure to hydrogen peroxide (250 μM) for 5 h. Shown is the heat map of TMRM fluorescence/well and scales of fluorescent intensity. Mean TMRM fluorescence/cell ± SD was significantly higher with treatment of CM-20 at 48 h (*P* = *0.0096*) and 72 h (*P* = *0.0021*).

Next, we examined the protective effect of CM-20 on mitochondrial activity under oxidative stress. Under exposure to hydrogen peroxide, the heat map of TMRM fluorescence in microplate dRPE cells pretreated with CM-20 showed stronger signals than the non-treated controls (Fig. 5). Further analysis of mean TMRM fluorescence/cell between CM-20 pretreated and nontreated controls also showed a significant difference. The protective effect of CM-20 on mitochondrial activity was related to the level of oxidative stress. At 250 μM (or lower) of hydrogen peroxide, mitochondrial activity was protected. At a higher concentration of hydrogen peroxide (> 500 μM), no obvious protective effect of CM-20 was observed.

## 4. DISCUSSION

In this report, we found novel bioactivity of an FDA-approved GPCR antagonist that could function as a mitochondrial modulator in RPE cells. GPCRs are a major class of drug targets for neurodegenerative diseases in the central nervous system [25 – 27]. Mitochondria have been considered as a therapeutic target for many prevalent diseases, including AMD [28, 29]. Treatment with CM-20 stimulated mitochondrial membrane potential in a dose-dependent manner. Mitochondrial membrane potential is the most reliable indicator for mitochondrial function in bioenergy production during oxidative metabolism [22,, 30]. Using a multiplexed live-cell mitochondrial imaging technique, we provided additional supporting experimental evidence to validate the findings from the dose-response study. While the direct use of ARPE-19 cells can be an alternative model for in vitro drug/compound testing, we chose to differentiate the cells to more closely simulate native RPE cells. The differentiated ARPE-19 cells showed typical cobblestone morphology of native RPE cells (Fig. 1A). There was no cellular toxicity in the tested 0–25 μM dose range of the compound. This is not surprising and expected given its approved clinical use in humans with an acceptable safety profile. Further, stimulation of MMP by the compound did not cause cellular damage in the cell viability assay. Treatment with CM-20 increased MMP in a dose and time-dependent manner (Fig. 1B). This finding is consistent with the secondary multiplexed mitochondrial imaging assays with an optimal concentration of the compound (Fig. 2). We did not present the data of TMRM-EC50 for CM-20, as the calculated value was affected by the amount of TMRM used in the assays and the treatment duration with CM-20. In fact, different conditions in cell-based assays can affect the EC50 value of the testing drug [31]. The intensity of TMRM fluorescence can be an indicator of mitochondrial abundance [32]. An increase in TMRM fluorescence under CM-20 treatment suggests that the protective mechanism of this drug compound may involve boosting mitochondrial biogenesis.

Findings from our work indicate that mitochondria in human RPE cells are vulnerable to functional damage under oxidative stress (Fig. 4). Hydrogen peroxide we used in the induction of RPE oxidative stress is an endogenous oxidative stressor in the retina [33]. In our study, we observed a significant loss of mitochondrial activity even under low to moderate levels of oxidative stress. However, the cells have been found resistant to oxidative stress-induced cell death. This finding is consistent with a previous report showing that differentiated ARPE-19 cells become more resistant to oxidative stress [34]. Pre-treatment with CM-20 showed rescuing effects on MMP under oxidative stress (Fig. 5). At the single cell level, mean TMRM fluorescence seemed higher under oxidative stress than that under low serum conditions (Fig. 5). This may result from the difference in cell density in microplates between experiments but does not necessarily indicate a higher mean TMRM fluorescence per cell level under oxidative stress.

A limitation of the study is the unknown effect of the compound on mitochondrial respiration. How would the compound affect mitochondrial respiration under resting conditions and under oxidative stress? A study on mitochondrial respiration would provide additional information to assess the utility of this drug for mitochondrial therapy.

## CONCLUSION

We have provided experimental evidence showing that a marketed drug of GPCR antagonist used to treat hypertension and heart disease modulates mitochondrial function and protects mitochondria from functional damage under oxidative stress in RPE cells. This drug compound possesses previously unappreciated dual targeting activities of both GPCR and mitochondria. It offers the potential in polypharmacological therapy for retinal degenerative diseases, such as AMD, via repurposed applications.

## LIST OF ABBREVIATIONS

RPE: Retinal Pigment Epithelial
dRPE: differentiated retinal pigment epithelial
MMP: Mitochondrial Membrane Potential
TMRM: Tetramethylrhodamine Methyl Ester
GPCR: G-protein Coupled Receptor
ER: Estrogen Receptor
FBS: Fetal Bovine Serum
